# Histone antibodies as a biomarker of uveitis in JIA

**DOI:** 10.1186/1546-0096-12-S1-O4

**Published:** 2014-09-17

**Authors:** Ellen B Nordal, Lillemor Berntson, Kristiina Aalto, Anders Fasth, Troels Herlin, Susan Nielsen, Suvi Peltoniemi, Marek Zak, Marite Rygg

**Affiliations:** 1 Department of Pediatrics, University Hospital of North Norway; 2 Department of Clinical Medicine, The Arctic University of Norway, Tromsø, Norway; 3 Department of Pediatrics, Uppsala University Hospital, Uppsala, Sweden; 4 Department of Pediatrics, Children’s Hospital, Helsinki University Hospital, Helsinki, Finland; 5 Department of Pediatrics, University of Gothenburg, Gothenburg, Sweden; 6 Department of Pediatrics, Århus University Hospital, Århus, Denmark; 7 Pediatric Rheumatology Department, Pediatric Clinic II, Copenhagen University Hospital, Rigshospitalet, Copenhagen, Denmark; 8 Department of Laboratory Medicine, Children’s and Women’s Health, Norwegian University of Science and Technology, Trondheim, Norway; 9 Department of Pediatrics, St Olavs Hospital, Trondheim, Norway

## Introduction

Uveitis is the most common extraarticular manifestation of juvenile idiopathic arthritis (JIA). The nature of JIA-associated uveitis is often insidious and asymptomatic, and baseline predictors can aid early diagnosis of eye disease for prompt and adequate treatment. Antihistone antibodies (AHA) are among subtypes of antinuclear antibodies (ANA) identified in children with JIA. An association between early-onset JIA, oligoarthritis and uveitis is shown in some studies, and we have previously shown that AHA is a significant predictor of chronic uveitis in a Norwegian JIA cohort. New interest for histones has emerged because epigenetic alterations of these DNA-binding molecules may be involved in the pathological processes of autoimmunity.

## Objectives

The aim of the study was to analyze presence of AHA in children with JIA with and without uveitis. We also wanted to compare AHA to previously described predictors of uveitis, such as early-onset arthritis, presence of ANA, oligoarticular ILAR category, and female gender.

## Methods

Consecutive cases of JIA from defined geographical areas of Denmark, Finland, Norway and Sweden with disease onset in 1997 to 2000 were included and followed for >7 years in a multi-center cohort study. Clinical information on joint and eye disease was prospectively collected in this longitudinal study aimed to be as close to population-based as possible. ANA-IF was analyzed twice >3 months apart in local laboratories. Serum samples taken early after disease onset were analyzed for AHA IgG/IgM in an enzyme immunoassay (Varelisa EIA Pharmacia Diagnostics) for the Danish and Swedish cohort. No serum samples were available in the Finnish cohort, and antihistone analyses in the Norwegian cohort has previously been published.

## Results

Uveitis occurred in 21.7 % of the 424 children with regular ophtalmologic follow-up, among the total cohort of 500 children. In the Danish and Swedish sub-cohort of 189 children, 132 had available serum samples. Significant predictors of chronic uveitis were onset of arthritis ≤7 years (OR 2.6 (1.5-4.7)), presence of antihistone antibodies (AHA ELISA IgM/IgG >15 U/ml) (OR 4.3 (1.5-12.3)), antinuclear antibodies (ANA IF) (OR 2.0 (1.2-3.5)), and presence of both ANA and AHA (OR 9.1 (2.5-32.9). Gender and oligoarticular onset category did not reach significance as predictors of uveitis. Mean AHA ELISA IgM/IgG was significantly higher in the children with uveitis (19.2 U/ml) than in the non-uveitis group (10.3 U/ml) (p=0.002).

## Conclusion

AHA, ANA and early-onset arthritis were significant predictors of chronic uveitis in the Nordic JIA cohort study. The strongest predictor was presence of both ANA and AHA. The present results in the Swedish and Danish sub-cohorts confirm previous findings in the Norwegian sub-cohort.

## Disclosure of interest

None declared.

